# Clinically acquired new challenging dataset for brain SOL segmentation: AJBDS-2023

**DOI:** 10.1016/j.dib.2023.109915

**Published:** 2023-12-07

**Authors:** Javaria Amin, Muhammad Almas Anjum, Nadia Gul, Muhammad Sharif, Seifedine Kadry

**Affiliations:** a Department of Computer Science, University of Wah, Wah Cantt, Pakistan; b National University of Technology, Islamabad, Pakistan; c Nadia Gul, FCPS Diagnostic Radiology, Consultant Radiologist POF hospital and Associate Professor of Radiology Wah Medical College, Wah Cantt. Pakistan; d Department of Computer Science, COMSATS University Islamabad, Wah Campus, Pakistan; e Department of Applied Data Science, Noroff University College, Kristiansand, Norway; f Department of Electrical and Computer Engineering, Lebanese American University, Byblos, Lebanon; g MEU Research Unit, Middle East University, Amman 11831, Jordan

**Keywords:** Almas Javeria Brain dataset (AJBDS)-2023, Multiplanar multi-sequential images (MPMSI), Segmentation, Algorithm

## Abstract

Space-occupying lesions (SOL) brain detected on brain MRI are benign and malignant tumors. Several brain tumor segmentation algorithms have been developed but there is a need for a clinically acquired dataset that is used for real-time images. This research is done to facilitate reporting of MRI done for brain tumor detection by incorporating computer-aided detection. Another objective was to make reporting unbiased by decreasing inter-observer errors and expediting daily reporting sessions to decrease radiologists’ workload. This is an experimental study. The proposed dataset contains clinically acquired multiplanar, multi-sequential MRI slices (MPMSI) which are used as input to the segmentation model without any preprocessing. The proposed AJBDS-2023 consists of 10667 images of real patients imaging data with a size of 320*320*3. Acquired images have T1W, TW2, Flair, T1W contrast, ADC, and DWI sequences. Pixel-based ground-truth annotated images of the tumor core and edema of 6334 slices are made manually under the supervision of a radiologist. Quantitative assessment of AJBDS-2023 images is done by a novel U-network on 4333 MRI slices. The diagnostic accuracy of our algorithm U-Net trained on AJBDS-2023 was 77.4 precision, 82.3 DSC, 87.4 specificity, 93.8 sensitivity, and 90.4 confidence interval. An experimental analysis of AJBDS-2023 done by the U-Net segmentation model proves that the proposed AJBDS-2023 dataset has images without preprocessing, which is more challenging and provides a more realistic platform for evaluation and analysis of newly developed algorithms in this domain and helps radiologists in MRI brain reporting more realistically.

Specifications Table

**Table:** 

Subject	Dataset article
Specific subject area	MRI Brain with space-occupying lesions
Type of data	Image
How the data were acquired	Data was collected from Dec 2018 to Jan 2023 in the radiology department of POF Hospital Wah Cantt. Ethical approval is taken from the head of the institution. Patient personal identity is not shown.
Data format	Raw
Description of data collection	A total of 10667 MRI brain slices of 33 randomly selected patients who had benign and malignant brain tumors. MRI brains were already reported by consultant radiologists. 6334 out of 10667 of 24 patients were used to make the AJBDS-2023 dataset. A U-Net algorithm is trained on the proposed dataset. Then 4333 slices of 9 patients were used to test the U-Net algorithm, to find the diagnostic accuracy of this dataset. Both male and female patients between the ages of 7 to 83 years are included in the study. All included patients had solitary benign or malignant brain tumors. Patients with multiple SOL brains were excluded. All MRIs were done in the Radiology department of POF Hospital on a 1.5 Tesla Seimens wide bore machine. Proposed dataset includes T1W, T2W, Flair, T1W-contrast, ADC, and DWI sequences in axial, coronal, and sagittal planes with skull bones. The image size of each patient slice is 320*320*3 pixels. The images are in jpg format.
Data source location	Pakistan Ordinance Factory (POF) Hospital, Wah Cantt, Pakistan
Data accessibility	https://data.mendeley.com/datasets/gmr8yyn77c/1 **Amin, JAVARIA; Anjum, Muhammad Almas (2023), “AJBDS-2023”, Mendeley Data, V2,**doi:10.17632/gmr8yyn77c.2

## 1 Value of the Data

**Fig. 1:**
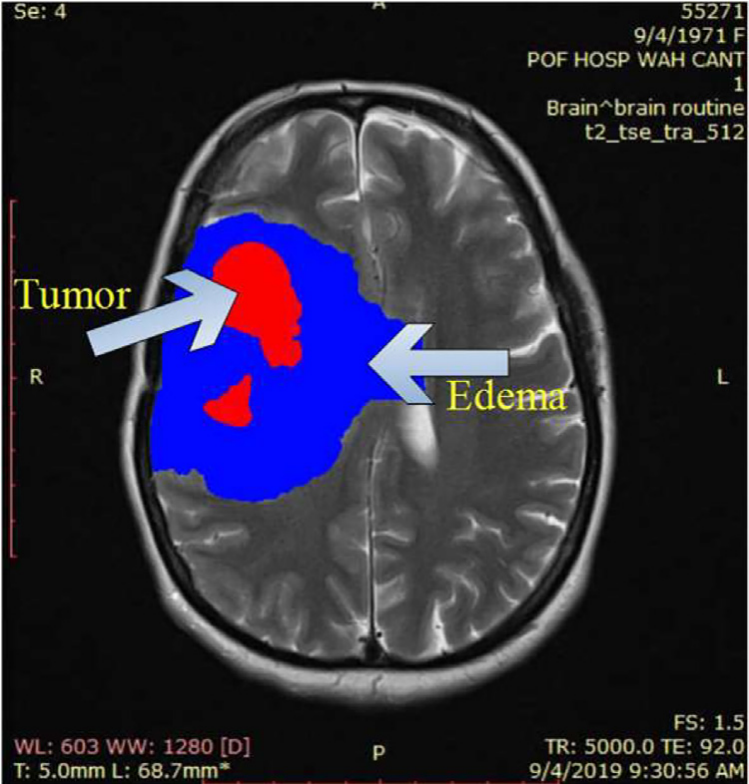
Shows the labels (the blue region denotes the edema, and the red region represents the tumor.

• SOL brain is mostly primary or metastatic malignant brain tumors, but these may be benign like abscesses, cysts, or hematoma.
• Worldwide multiple MRI brain image segmentation datasets have been developed. These datasets use advanced computer algorithms and machine learning techniques to automatically segment and classify different brain tissue types and abnormalities in MRI images.
• The existing methods have been evaluated on the available datasets like the Challenge series [1], [2], [3]. Different versions of available datasets provide pre-processed data with the subtraction of skull bones which makes it far different from the real-time acquired imaging data.
• This problem is solved by processing the multi-sequential MRI data of a real patient with ground masks. The proposed dataset contains more challenging clinical features (i.e., including skull bones, and marking the complete tumor and edema region) as shown in Fig. 1, which are not available in the previously publicly available benchmark datasets.

All MRI brain cases used in this study were initially reported by consultant radiologists of POF Hospital. Then color and binary mapping was done on real MRI images under the direct supervision of a consultant radiologist which included brain along with skull bones without preprocessing, which makes this data set more realistic to train new algorithms. We tested the diagnostic yield of our dataset on the U-Network algorithm which gave satisfactory diagnostic accuracy. At the initial stage of this research, we included images with only solitary brain SOL/tumors. In future series of this dataset, we will do research on multiple brain SOL/tumors and will do multicenter data collection to find trends of different brain tumors in different ethnicities.

## 2 Objective

The primary objective of this study is to develop a real patient brain tumor segmentation dataset without any standard pre-processing to evaluate the brain tumor detection algorithms on real patient data. Secondly, it will also facilitate the research in this domain by providing a benchmark for researchers to test their algorithms by using directly available clinical data (MRI) that will provide help in early diagnosis and patient management.

This research is done to facilitate reporting of MRI done for brain tumor detection by incorporating computer-aided detection. Another objective was to make reporting unbiased by decreasing inter-observer errors and expediting daily reporting sessions to decrease radiologists’ workload.

## 3 Data Description

It is an experimental study. Data was collected from Dec 2018 to Jan 2023 in the radiology department of POF Hospital Wah Cantt. Ethical approval is taken from the head of the institution (Commandant POF Hospital Wah Cantt). A total of 10049 slices of 32 randomly selected patients who had benign and malignant brain tumors on MRI brains already reported by consultant radiologists are included. 6334 out of 10667 of 24 patients were used to make the AJBDS-2023 dataset. A U-Net algorithm is trained on the proposed dataset. Then 4333 slices of 8 patients were used to test the U-Net algorithm, to find the diagnostic accuracy of this dataset. Both male and female patients between the ages of 7 to 83 years are included in the study. All included patients had solitary benign or malignant brain tumors. Patients with multiple SOL brains were excluded. All MRIs were done in the Radiology department of POF Hospital on a 1.5 Tesla Seimens wide bore machine. The proposed dataset AJBDS-2023 includes T1W, T2W, Flair, T1W-contrast, ADC, and DWI sequences in axial, coronal, and sagittal planes with skull bones. The image size of each patient slice is 320*320*3 pixels. The images are in jpg format. Table 1 shows a detailed description of each case.

**Table 1:** Description of cases of POF hospital.

Patients	Age	Sequences	Total images
P#1	11 Year(s)	T1W, TW2, Flair, T1W contrast, ADC, and DWI	341
P #2	47 Year(s)	T1W, TW2, Flair, T1W contrast, ADC, and DWI	198
P #3	64 Year(s)	T1W, TW2, Flair, T1W contrast, ADC, and DWI	298
P #4	63 Year(s)	T1W, TW2, Flair, T1W contrast, ADC, and DWI	333
P #5	66 Year(s)	T1W, TW2, Flair, T1W contrast, ADC, and DWI	356
P #6	49 Year(s)	T1W, TW2, Flair, T1W contrast, ADC, and DWI	177
P#7	56 Year(s)	T1W, TW2, Flair, T1W contrast, ADC, and DWI	218
P#8	57 Year(s)	T1W, TW2, Flair, T1W contrast, ADC, and DWI	255
P#9	57 Year(s)	T1W, TW2, Flair, T1W contrast, ADC, and DWI	134
P#10	36 Year(s)	T1W, TW2, Flair, T1W contrast, ADC, and DWI	320
P#11	56 Year(s)	T1W, TW2, Flair, T1W contrast, ADC, and DWI	371
P#12	7 Year(s)	T1W, TW2, Flair, T1W contrast, ADC, and DWI	322
P#13	64 Year(s)	T1W, TW2, Flair, T1W contrast, ADC, and DWI	316
P#14	55 Year(s)	T1W, TW2, Flair, T1W contrast, ADC, and DWI	80
P#15	7 Year(s)	T1W, TW2, Flair, T1W contrast, ADC, and DWI	342
P#16	33 Year(s)	T1W, TW2, Flair, T1W contrast, ADC, and DWI	175
P#17	29 Year(s)	T1W, TW2, Flair, T1W contrast, ADC, and DWI	208
P#18	60 Year(s)	T1W, TW2, Flair, T1W contrast, ADC, and DWI	183
P#19	32 Year(s)	T1W, TW2, Flair, T1W contrast, ADC, and DWI	322
P#20	45Year(s)	T1W, TW2, Flair, T1W contrast, ADC, and DWI	312
P#21	48 Year(s)	T1W, TW2, Flair, T1W contrast, ADC, and DWI	291
P#22	66 Year(s)	T1W, TW2, Flair, T1W contrast, ADC, and DWI	275
P#23	76 Year(s)	T1W, TW2, Flair, T1W contrast, ADC, and DWI	272
P#24	72 Year(s)	T1W, TW2, Flair, T1W contrast, ADC, and DWI	235
P#25	39 Year(s)	T1W, TW2, Flair, T1W contrast, ADC, and DWI	605
P#26	14 Year(s)	T1W, TW2, Flair, T1W contrast, ADC, and DWI	317
P#27	67 Year(s)	T1W, TW2, Flair, T1W contrast, ADC, and DWI	459
P#28	58 Year(s)	T1W, TW2, Flair, T1W contrast, ADC, and DWI	480
P#29	51 Year(s)	T1W, TW2, Flair, T1W contrast, ADC, and DWI	441
P#30	47 Year(s)	T1W, TW2, Flair, T1W contrast, ADC, and DWI	467
P#31	31 Year(s)	T1W, TW2, Flair, T1W contrast, ADC, and DWI	483
P#32	55 Year(s)	T1W, TW2, Flair, T1W contrast, ADC, and DWI	618
P#33	83 Year(s)	T1W, TW2, Flair, T1W contrast, ADC, and DWI	463

A detailed description of the patients (Table 1) where the first column gives the total number of patients, the second column shows the age of the patient which is 7 to 83 Years, whereas the third column presents sequences of MRI slices i.e., T1W, TW2, Flair, T1W contrast, ADC, DWI. The fourth column shows the total number of [healthy + core tumor, edema] slices.

Four labels such as tumor core, necrosis, edema, and enhanced core tumor have been used in the proposed dataset. The tumor core refers to all parts of the tumor except edema, whereas the active tumor core includes only the enhanced part of the tumor. The visual structure of the SOL brain is defined as:

1. T2 scans are largely used to segment the “edema”. FLAIR is used to assess edema extension and distinguish it from ventricles as well as from other fluid regions.
2. Isointense tumors are difficult to see in plain studies same are visible on the T1C sequence, while inhomogeneous brain tumors are visible in the T1 sequence without contrast.
3. The fluid-filled non-enhancing core is known as necrosis inside the enhanced rim which is visible on T1C images.
4. The non-enhancing solid core represents the part of the gross core of the tumor excluding enhanced solid tumor and fluid-filled necrotic core structure.
5. The area of diffusion restriction in the tumor core represents blood flow restriction on DWI and ADC sequences. It appears hyperintense on DWI and correspondingly hypointense on ADC sequence.

## 4 Experimental Design, Materials, and Methods

**Fig. 2:**
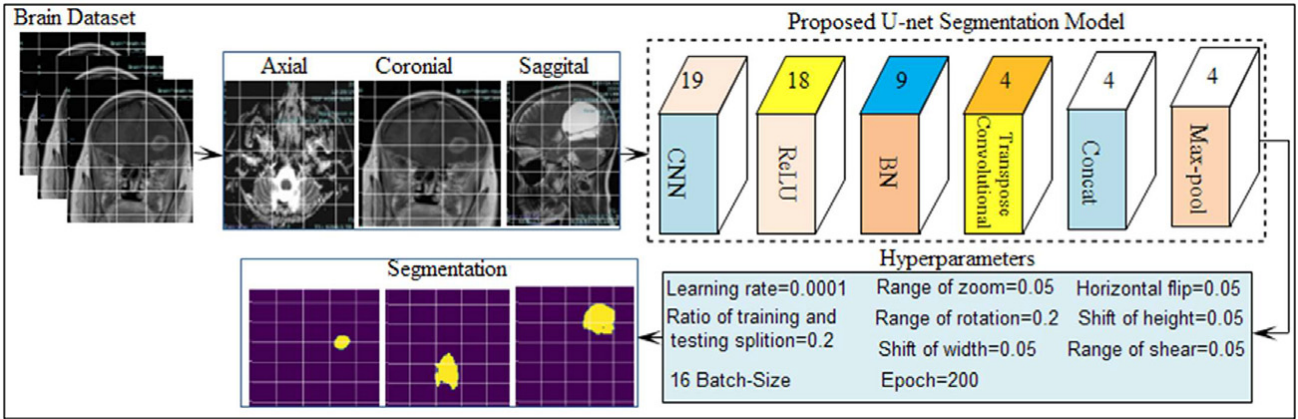
Design of the proposed study [SOL brain of axial, coronial and sagittal views images of AJBDS-23 dataset are passed to the proposed U-net model for segmentation].

In the proposed dataset annotating protocols have been used to visualize the structure of both edema and tumor region. In this process, “Adobephotoshop2019” has been used for the creation of ground masks. Clinical imaging data has been obtained in the DICOM format and converted into a jpg extension using the “Radiant DICOM” Viewer. After the jpg conversion, the tumor/edema region has been marked with careful examination of multi-sequential MRI slices. The tumor region was marked in red while the edema region was in blue. After color annotation, Adobe Photoshop 2019 was used for the creation of binary ground masks. In binary mask creation, the pen tool is used because it helps in the extraction of smooth boundary pixels. The feather's radius of 0, 1, and 2 Pixels is selected because brain lesions appear round. MPMSI scans are used to address the segmentation of core tumor and edema from T1/T2 weighted FLAIR, ADC, and DWI. Ground truth masks of 24 MPMSI slices have been created by and verified by classified radiologists. For the evaluation of AJBDS-23, a segmentation algorithm is trained on the proposed dataset and then tested on 4333 slices of 9 patients. Researchers can utilize the AJBDS-2023 dataset for algorithm training and testing, comparing their scientific experimentation and reporting their results to discuss the potential difference. The tumor region has been segmented by applying a fusion of staple of top-Brats ranked algorithms such as deep media, U-network, and deep scans in BRATS-2021 [4]. However, MPMSI scans of proposed AJBDS-2023 are provided with raw images of T1-W, T-W2, Flair, T1W-contrast, ADC, and DWI sequences, which are acquired from a local hospital in Pakistan. No standard pre-processing algorithms have been applied to AJBDS MPMSI scans. The skull region is included in the AJBDS-2023 to make it closer to the real scans. The U-net segmentation model is designed as shown in Fig. 2, which is trained on the proposed AJBDS-2023 dataset.

In the proposed dataset, different types of malignant and benign brain lesions were studied. Malignant masses include gliomas like GBM, Astrocytoma, Brain stem gliomas and Anaplastic oligodendroglioma. Other malignant brain tumors include brain metastases, pituitary macroadenoma, medulloblastoma, CP angle Schwannoma, and malignant meningioma. Benign tumors include Choroidal fissure cysts, Porencephalic cysts, CP angle cysts, Neuroglial cysts, and Meningioma. The number of different types of lesions is mentioned in Table 2.

**Table 2:** Different types of the brain SOL.

Type	Number
Abscess	2
Glioblastoma (GBM)	7
Astrocytoma	5
Brain stem gliomas	1
Brain metastases	2
Choroidal fissure cysts	1
Porencephalic cyst	1
CP angle cyst	1
Pituitary macroadenoma	3
Cp angle Schwannoma	2
Neuroglial Cyst	1
Medulloblastoma	2
Meningioma	4
Anaplastic oligodendroglioma	1

In this work studied total of 33 cases in which SOL 21 are intra axial, while 12 SOL are extra axial. 28 out of 33 tumors were malignant and 5 were benign. The percentage and size of the benign and malignant tumors are provided in Table 3.

**Table 3:** Percentage and size of tumors in the AJBDS-23 dataset.

Cases (33)	Intra axial	Extra axial	Min size	Max size
Malignant tumor 28 (84.8 %)	21 (63.6 %)	12 (36.3 %)	Less than 3	8 cm
Benign tumor 5 (15.1 %)			Less than 3	6.4 cm

The diagnostic accuracy is computed in terms of predictive values (PV), dice similarity coefficient (DSC), specificity (SP), sensitivity (SE), and confidence interval (CI) as mentioned in Table 4.

**Table 4:** Proposed segmentation outcomes.

Phases	PV	DSC	SP	SE	CI
Training	80.6	90.0	89.1	95.2	95.4
Validation	78.1	88.2	89.1	94.5	91.5
Testing	77.4	82.3	87.4	93.8	90.4

Table 5 depicts the results comparison of the existing BRATS dataset to the proposed AJBDS-2023 dataset.

**Table 5:** Comparison of the Tumor core in terms of DSC.

Refs#	Year	BRATS-2019	BRATS-2020	BRATS-2021	DSC
[5]	2023	✓			83.0
[6]	2023		✓		83.5
[6,7]	2022 2023			✓	85.3 84.0
**Proposed AJBDS-2023**					82.3

**Fig. 3:**
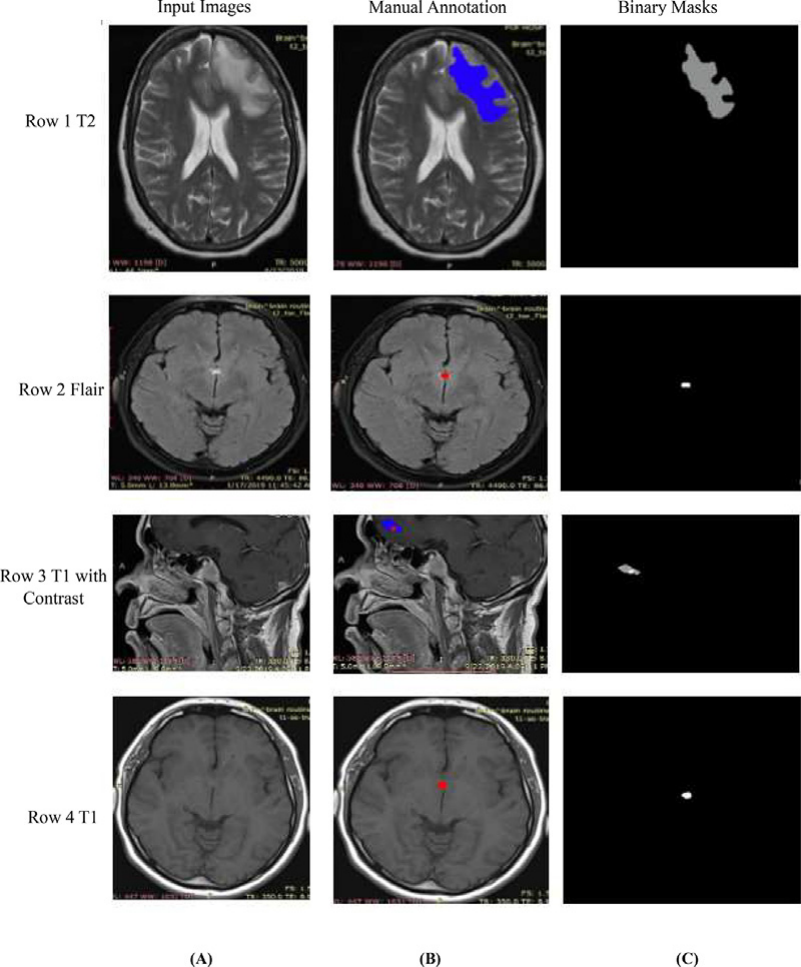
(A) input images (B) manual annotations by radiologists (C) binary segmentation [In which shows the labels (Row 1) axial view of T2 shows edema (blue), (Row 2) axial T2 Flair shows solid tumor component in red color, (Row 3) (T1 with contrast) red core tumor with necrotic cells and solid tumor (red) color, edema (blue), (Row 4), T1, axial view red shows the solid tumor].

The proposed dataset provides multi-labels of SOL which are manually marked by classified radiologists and shown in Fig. 3.

## Limitations

All tumor cases included in this study are solitary brain tumors, so the diagnostic accuracy of algorithms trained on this data set may not be the same for multiple brain tumors like multifocal abscesses and metastasis. This study is done on a limited number of patients in a single hospital, so this dataset may be improved if it will be extended to multiple centers and will include patients of multiple ethnicities in different countries.

This dataset may train very useful algorithms which can expedite MRI brain tumor reporting and help radiologists by decreasing their workload. Moreover, it will be able to get standardized results without radiologist bias and inter-observer errors. This can further help to find trends of neoplastic brain diseases in a certain area, race, and socioeconomic status.

**Fig. 4:**
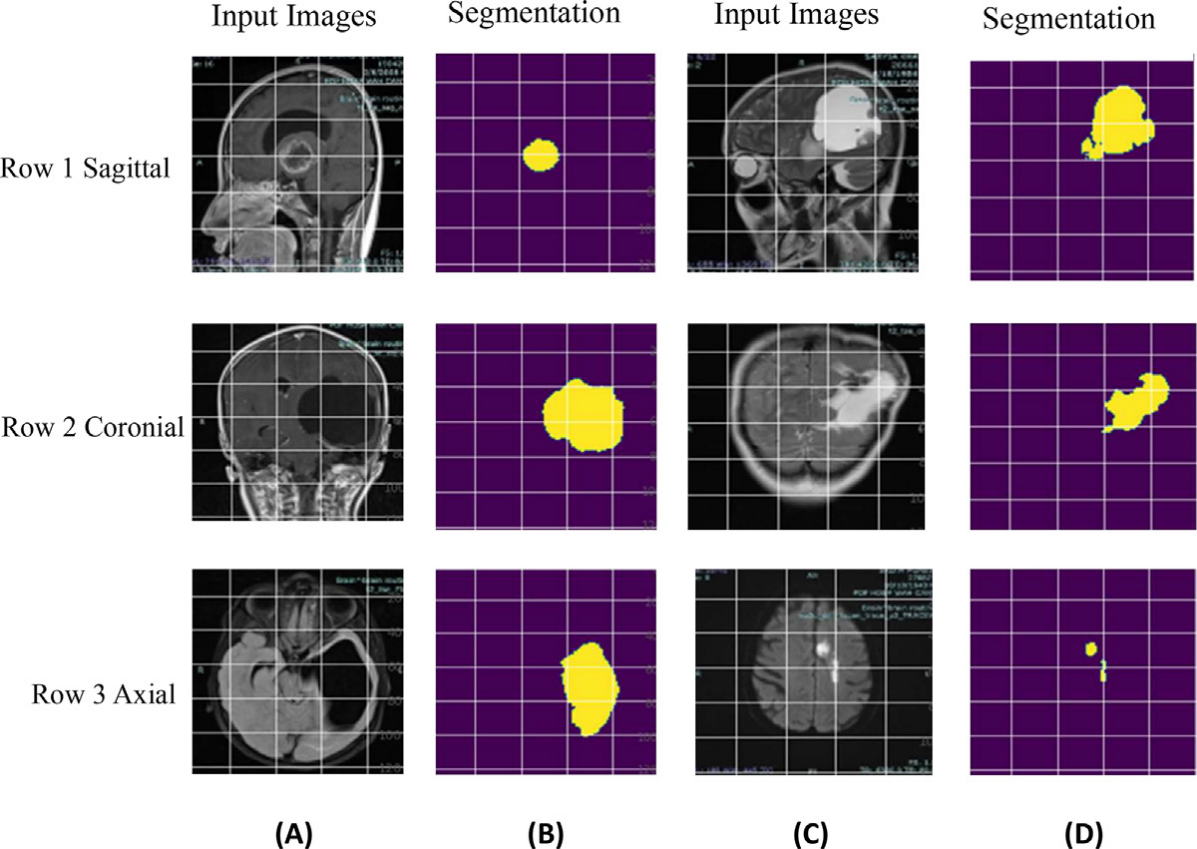
Segmentation results of the proposed method Column of **(A) (C)** sagittal, coronial, axial views and Column of **(B) (D)** segmented region [In which Row 1, Row 2, Row 3 shows Sagittal, Coronial and Axial respectively].

At the testing phase, the proposed U-net model more accurately segments the SOL brain as shown in Fig. 4.

## Ethics Statements

This dataset used MRI brain images retrospectively, already available on PACS of POF hospital so informed consent of patients could not be taken. However, we respected the patient's personal identity i-e name, MR no, age, gender is exluded in the included images. Also we took permission from the head of institution to use this data for research purpose and permission letter is attached.

The permission letter is attached. The full study protocol can be accessed through the following link: https://data.mendeley.com/datasets/gmr8yyn77c/1.

## CRediT authorship contribution statement

**Javaria Amin:** Writing – original draft, Conceptualization, Software, Visualization, Validation, Methodology. **Muhammad Almas Anjum:** Writing – review & editing. **Nadia Gul:** Data curation. **Muhammad Sharif:** Supervision. **Seifedine Kadry:** Funding acquisition.

## Data Availability

AJBDS-2023 (Original data) (https://data.mendeley.com/datasets/gmr8yyn77c/1)
